# Feasibility of a Portable Electronic Nose for Detection of Oral Squamous Cell Carcinoma in Sudan

**DOI:** 10.3390/healthcare9050534

**Published:** 2021-05-03

**Authors:** Nazar Mohamed, Rens van de Goor, Mariam El-Sheikh, Osman Elrayah, Tarig Osman, Elisabeth Sivy Nginamau, Anne Christine Johannessen, Ahmed Suleiman, Daniela Elena Costea, Kenneth W. Kross

**Affiliations:** 1 Center for Cancer Biomarkers (CCBIO) and Gade Laboratory for Pathology, Department of Clinical Medicine, University of Bergen, P.O. Box 7800, 5020 Bergen, Norway; nazar.mohamed@uib.no (N.M.); tarig.osman@uib.no (T.O.); elisabeth.nginamau@uib.no (E.S.N.); anne.johannessen@uib.no (A.C.J.); 2 Center for International Health (CIH), University of Bergen, P.O. Box 7800, 5020 Bergen, Norway; 3 Department of Oral and Maxillofacial Surgery and Department of Basic Sciences, University of Khartoum, P.O. Box 321, 11111 Khartoum, Sudan; mariam.elhadi@uofk.edu (M.E.-S.); osman.elrayah@uofk.edu (O.E.); newsulaiman@uofk.edu (A.S.); 4 Department of Otolaryngology—Head and Neck Surgery, Bernhoven Hospital, P.O. Box 707, 5400 AS Uden, The Netherlands; rens.vande.goor@mumc.nl; 5 Department of Otolaryngology—Head and Neck Surgery, Maastricht University Medical Centre, P. Debyelaan 25, 6229 HX Maastricht, The Netherlands; 6 Department of Pathology, Haukeland University Hospital, Jonas Lies vei 65, N-5020 Bergen, Norway; 7 Policlinique Saint Odilon, 32 Rue Professeur Etienne Sorrel, 03000 Moulins, France

**Keywords:** electronic nose, cancer, oral, diagnosis, screening, toombak

## Abstract

Background: Oral squamous cell carcinoma (OSCC) is increasing at an alarming rate particularly in low-income countries. This urges for research into noninvasive, user-friendly diagnostic tools that can be used in limited-resource settings. This study aims to test and validate the feasibility of e-nose technology for detecting OSCC in the limited-resource settings of the Sudanese population. Methods: Two e-nose devices (Aeonose™, eNose Company, Zutphen, The Netherlands) were used to collect breath samples from OSCC (*n* = 49) and control (*n* = 35) patients. Patients were divided into a training group for building an artificial neural network (ANN) model and a blinded control group for model validation. The Statistical Package for the Social Sciences (SPSS) software was used for the analysis of baseline characteristics and regression. Aethena proprietary software was used for data analysis using artificial neural networks based on patterns of volatile organic compounds. Results: A diagnostic accuracy of 81% was observed, with 88% sensitivity and 71% specificity. Conclusions: This study demonstrates that e-nose is an efficient tool for OSCC detection in limited-resource settings, where it offers a valuable cost-effective strategy to tackle the burden posed by OSCC.

## 1. Introduction

Disease-associated odor is an old phenomenon, which was first mentioned by Hippocrates of Kos (460–370 BC), who described “fetor oris” and “fetor hepaticus”. The interest for this phenomenon developed over time throughout Antoine Lavoisier studies in the 18th century [1,2]. Modern era of breathomics started in 1970s with the pioneering research of Linus Pauling on analyzing urine vapor utilizing gas chromatography [3]

Gas chromatography-mass spectrometry (GC-MS) is the gold-standard platform that identifies individual volatile organic compounds (VOCs) according to their physical features when compared to a reference library. Unfortunately, its clinical use is not feasible since it is not real-time, needs a long time for sample processing, relies on non-portable devices, and needs constant calibration for specific analytes. Other MS-hybrid methodologies were also proved to be useful [4,5]. 

Electronic noses are devices that also allow detection and identification of various volatile compounds or odors based on gas sensor arrays to simulate the function of the human olfactory system [6]. Concomitant with rapid developments in sensor technology, there is a plethora of electronic noses (e-noses) technologies that appeared lately [7], displaying different sensor chemistries [8,9,10,11,12,13]. As such, sensor arrays used in e-noses include many types of electrochemical sensors and different types of sensor-coating materials which are classified according to additive doping materials, the type and nature of the chemical interactions, the reversibility of the chemical reactions and running temperature. Those includes electrochemical (EC), metal oxide semiconductor (MOS), nondispersive infrared sensors (NDIR), thermal sensor, and photoionization sensor (PID) [14]. The most widely used class of gas sensors are MOSs. The selectivity of these sensors can be changed using different noble metals or by changing the operating temperature. They are very sensitive, robust, humidity resistant, and durable, although they can suffer from drift over time [7,15].

In addition to sensors, there are different transducers recording devices according to what they measure as output from the sensor, e.g., electrical measurement or optical measurement [10]. The output signal of a sensor in a gas sensor arrays, represents components of a vector, which is normally processed by feature extraction first, followed by preprocessing and normalization; then explanatory techniques are applied [8,16,17]. The measurement data from sensor arrays are multidimensional, therefore dimensionality reduction and classification methods are used to furthermore process the data [8]. The cross-reactive sensor arrays are usually coupled with a pattern recognition algorithm to detect VOC fingerprint patterns resulted from the combination of signals produced by different sensors [18]. 

E-nose technology can use exhaled breath VOC pattern analysis in order to detect specific pathological conditions in different hosts [6,19,20] and to create a prediction model. These VOCs are products of different metabolic processes, including cancer metabolism, that dissolve in the bloodstream and enter the respiratory tract through alveoli [21]. It has been demonstrated that specific VOCs for head and neck squamous cell carcinoma (HNSCC), including a subset of oral squamous cell carcinoma (OSCC), can be detected with e-nose technology using pattern recognition in which a-specific sensors are combined with machine learning [22]. Using an artificial neural network (ANN) technique, these individual patterns can be transferred to a model for diagnosing HNSCC, including OSCC.

While significant improvement has been made in the detection and treatment of most malignancies, the prognosis of OSCC has remained relatively unchanged [23,24]. In Sudan, OC is the sixteenth most common cancer [25], with an incidence of 775 cases per year [26], most cases presenting at stages III or IV [27].

Full diagnosis of OSCC requires a specialized setting where clinical examination is followed by contrast-enhanced computerized tomography (CT) and/or magnetic resonance imaging (MRI) in addition to the histopathological examination of biopsies, which is essential for proper diagnosis [28]. Current diagnostic tools for OSCC are invasive and expensive, posing difficulties in Sudan, where the specialized diagnostic centers are sparse and located in the main cities only. 

There, devices based on electronic nose (e-nose) technology could meet the need for low-cost, easy-to-use tools for diagnosis of OSCC at early stages, which could substantially prolong life expectancy and reduce the costs of treatment. The main advantage of the e-nose is its user-friendly design. This portable handheld device does not require specialized facilities or personnel. The e-nose could provide results in just 15 min after the patient starts exhaling through the device and if the device is connected to an internet network. However, the e-nose is not intended to replace the histopathological examination of oral biopsies, which should still be the gold standard for the final diagnosis and staging of OSCC [29]. It is conceived that it will help to reduce the workload demanded by the still invasive and time-consuming conventional biopsy procedure by better selection of malignant suspicious cases since it is well recognized that conventional oral examination (COE) is not predictive of histological diagnosis of oral lesions OSCC [30]. Add to that the inter- and intra-observer reliability in reporting [31], utilization of e-nose methodology is meant to be used in addition to COE for better selection of cases for biopsies, which will be of value particularly in the limited-resource settings of low-income countries such as Sudan.

The present study aimed to test the feasibility of e-nose technology for detecting OSCC in the limited-resource settings of the Sudanese population. Similar studies testing the feasibility of e-nose have been carried out on Western populations [32]. However, there are significant differences between the Sudanese and a Western population, not only in terms of the type of the tobacco consumed [33,34] but also in the availability of resources and health-care networks, differences justifying the present study.

## 2. Materials and Methods

### 2.1. Study Cohort

This study was conducted at Khartoum Teaching Dental Hospital in Khartoum, Sudan (tertiary care referral hospital), from July 2016 through October 2018. The study protocol (no. 2016/4) was approved by the Medical Ethics Committee at the Ministry of Health, Sudan, and all methods were performed in accordance with the relevant guidelines and regulations. Inclusion criteria were consecutive patients who had histologically confirmed primary OSCC (C00–C06) during that period and healthy non-cancer controls, who consented to participate in the study. As far as possible, the healthy controls were selected among age- and sex-matched individuals who visited the out-patient clinic of the same hospital for routine dental treatments. Exclusion criteria were being younger than 18 years of age, having any previous or current cancer diagnosis, any treatment for the current tumor or a history of cancer, and other histological types of lesions/tumors than OSCC. Six cases later confirmed histologically as adenocarcinoma (two), oral cavity aspergillosis (two), and verrucous hyperplasia (two) were excluded from further analysis. Tumor characteristics and medical history were collected from the clinical records. TNM stage was registered according to version 7.0 of the American Joint Committee on Cancer Guidelines. Information on current smokeless tobacco (toombak) use, smoking habits, and history of smoking was collected and reported in pack-years, with calculations for *toombak* consumption adjusted according to the average of manually prepared portions in Sudan [35]. Nonsmoking was defined as no smoking during the previous month. Fasting was defined as ingesting no food for the last 3 h and just water or clear tea without additives for the last hour. 

To obtain a reliable model valid for ANN analysis using Aethena software, the sample size was calculated using the software PASS 2020, v20.0.3 (SPSS Inc., Chicago, IL, USA). The minimum number of *n* = 62 would give a power of 0.9 (alpha 0.15 and beta 0.099). 

### 2.2. Study Design 

**Figure 1:**
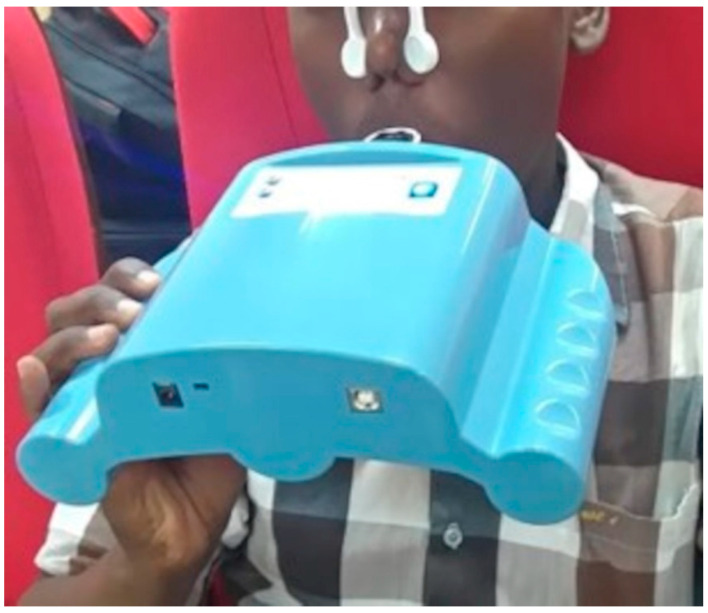
Taking a measurement from a patient using Aeonose device.

Before each measurement, patients were instructed to inhale and exhale through a disposable mouthpiece in the e-nose for 5 min. This mouthpiece contains a high-efficiency particulate arrestance (HEPA) filter, which protects the device from contamination, e.g., by bacteria and viruses. Patients were instructed to close their lips over the mouthpiece, and a nose clip was used to prevent nasal air passage (Figure 1).

Test runs of in- and exhalations were performed so that the patient could get acquainted with the device. Participants were breathing through a carbon filter to limit the possibility that environmental VOCs would tamper with the measurement. For the first 2 min, their lungs were rinsed with clean filtered air that passed through the carbon filter without passing the sensors and dead air space was removed. Afterwards, a valve was opened to allow exhaled air to interact with the sensors. The total measurement cycle lasted about 15 min, during which time the patient in- and exhaled through the device for 5 min. The remaining time was used to measure any low-concentrated VOCs inside the Tenax tube and to regenerate the sensors with clean filtered air (for details see van Hooren) [36].

Patients did not receive individual diagnostic results from the e-nose analysis. The results of these measurements did not influence the regular diagnostic work-up or treatment of the participants. All measurements were performed in the same room by the same operators.

### 2.3. Materials

The e-nose device (Aeonose^™^, The eNose Company, Zutphen, The Netherlands) contains micro hotplate metal-oxide-based sensors (AS-MLV sensors, Applied Sensors GmbH), which are heated and cooled in 32 steps with accurate regulation of temperature between 260 and 340 °C during the measurements. The change in the sensors’ conductivity follows the temperature-dependent reaction of VOCs from breathing air (redox reactions) and produces a unique VOC pattern, as previously described [36,37]. The measurement takes, in total, about 15 min, including 5 min spent on respiration with the patient holding the device (Figure 1). The next 10 min are used for sensor regeneration and detecting possible low-concentrated VOCs. For a more detailed discussion on this point-of-care device, see van Hooren et al., 2016 [36]. Two Aeonose^™^ devices (serial numbers 257 and 372) were used in this study to reduce any possible device-related confounding factors.

### 2.4. Statistical Analysis

Baseline group differences were determined using independent sample *t*-test, Fisher’s exact test, or Mann–Whitney U test according to data characteristics. Logistic regression has been also performed on the data including other clinical parameters such as gender, age, smoking (total pack-year), and toombak. All statistical analyses were performed using IBM Statistical Package for the Social Sciences (SPSS) for Windows, Version 25.0 (IBM Corp., Armonk, NY, USA). Logistic regression was done using forward-stepwise (conditional) method. The subject group (patient or control) was assigned as the dependent variable, while age, gender, toombak use, and smoking were assigned as independent variables. All categorical data were coded as 0 and 1. The predicted probability produced from each step in the model was used to generate the receiver operating characteristic curve, or ROC curves. 

During one measurement, 64 times 36 data points were recorded for each of the three sensors. A Tucker3-like solution for tensor decomposition was used to compress these data points of temperature, measurement cycle, and sensors [36]. In brief, the raw data points are normalized, per participant, between 0 and 1. Then spikes were removed by peak shaving. Fourier transformation was applied to compensate for the clean air signal, and then Fourier back transform was applied. Following that, the e-power was applied to all data points. NOx-sensor was only selected. Feature extraction was done to end up with only 19 element vector per participant. The resulted vectors were normalized between −1 and +1 (Supplementary Materials 1). 

The compressed data were pre-marked as either benign or malign and used to train the Artificial Neural Network (ANN). Data compression and ANN have been integrated into a proprietary software package (Aethena, The eNose Company, Zutphen, The Netherlands). A resilient backpropagating ANN training was executed for a number of data scaling options, resulting in multiple ANN options for separating benign from malignant conditions. The following parameters were used to train the ANN: Max Epoch: 5000, Max Retries: 25, Max Same Error: 30, Max Error Inc: 15, Minimal Error: 0.0005, Learn Rate: 0.0010, Alpha: 0.0500, Topology: 17 × 7. Data were cross-validated by the Leave-10%-Out method. This method prevents to a large extent the fitting of data on artefacts instead of on breath profile classifiers. 

To exclude possible block size device dependencies, no more than 5 consecutive measurements of healthy controls or patients with OSCC were allowed. Meaning that, e.g., a sixth consecutive OSCC patient measured is excluded when building the ANN for that specific model. This continues till a healthy control sample is measured. All patients excluded due to block size dependency were used to create the blinded group. 

The ANN model calculates a value between −1 and 1 for each patient, corresponding with the diagnosis for that patient. These data result in a ROC-curve for each ANN showing accuracy values that can be obtained by that specific model. These calculations were performed for each model separately and resulted in data on sensitivity, specificity, the area under the curve (AUC), and overall accuracy. The flow of data processing is described step by step in the Supplementary Materials 2. 

## 3. Results

### 3.1. Cohort Characteristics

A total of 84 patients with histologically confirmed OSCC and healthy controls (age range: 21–82; mean = 50.6 years; and median = 50.5 years) were included in the study. The collection of breath samples did not result in any adverse effects. Healthy controls were younger and reported more *toombak* consumption and/or smoking behavior than OSCC patients (Table 1). The localization of OSCC lesions was predominantly lower buccal or labial (51%); only 14.3% were localized on the tongue. Of all OSCC patients, 69.3% presented with locoregional lymph node metastases at the time of diagnosis. Only 4% of the OSCC cases presented at early stages; nearly all OSCC patients (85.8%) presented at a late stage.

### 3.2. Feasibility Analysis of E-Nose Measurements

**Figure 2:**
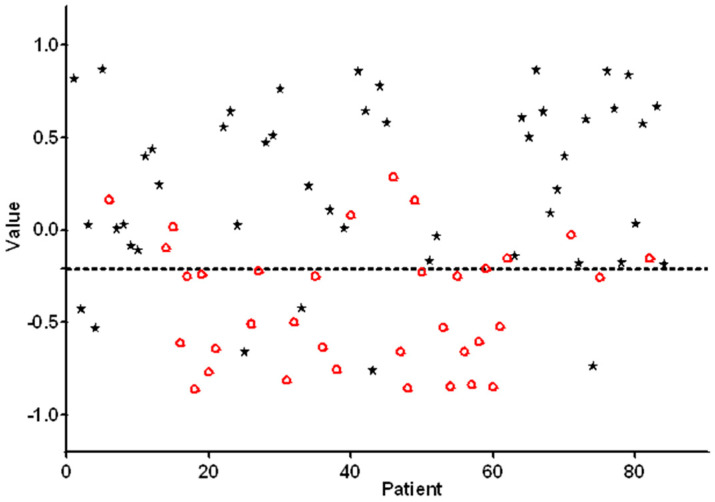
Individual e-nose values for each patient and control of the training set are plotted. Values > −0.21 are scored as being positive for oral squamous cell carcinoma (OSCC). The black asterisks represent patients with histopathologically confirmed OSCC. The red circles represent healthy controls.

A scatterplot of individual predicted values as calculated by ANN on the basis of e-nose measurements is presented in Figure 2. In order to obtain a high sensitivity combined with an acceptable specificity, the threshold was set to −0.21. Individual predicted values above this threshold were classified as positive, and the values below this threshold were classified as negative primary OSCC. Substantial variances in individual predicted values were observed; approximately 80% of the predictive values were located between −0.5 and 0.5.

**Figure 3:**
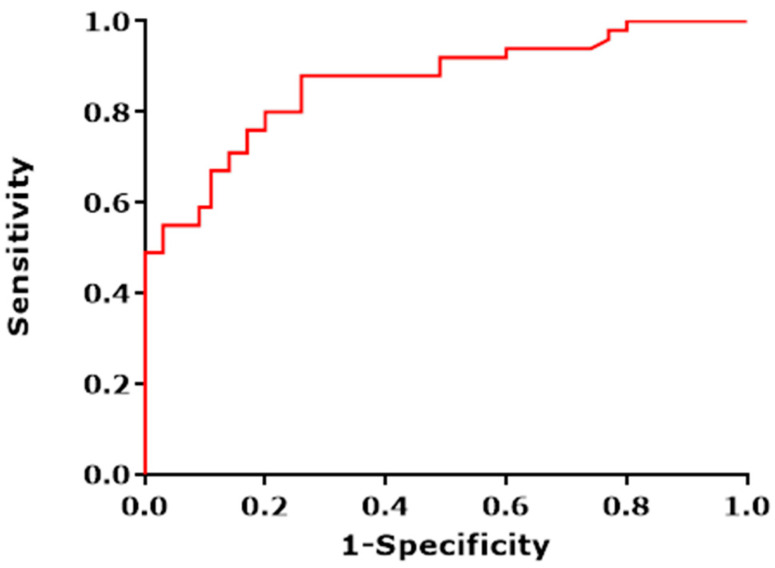
The ROC (receiver operating characteristic)curve depicting the different sensitivities and specificities with altered thresholds of both the best fit of data for cross validation (red line). Black line represents the line of no-discrimination. The area under the curve is 0.86.

**Figure 4:**
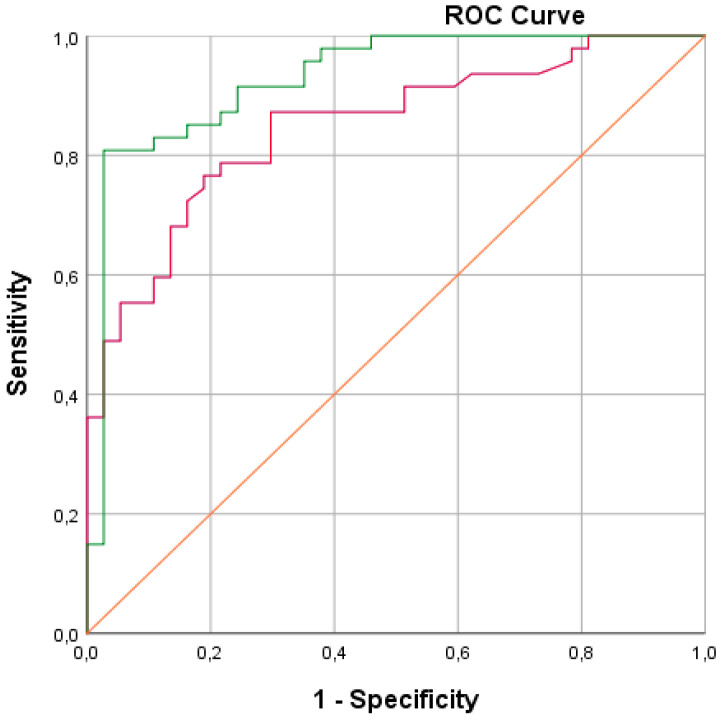
ROC (receiver operating characteristic) curves showing the best fit of data for cross-validation when including in the statistical analysis; the clinical parameters of gender, age, smoking (total pack-year), and toombak use (green line; the area under the green curve is 0.92) are compared to the best fit for cross-validation when only the e-nose test values are analyzed (red line).

We obtained a sensitivity (SE) of 88% with a corresponding specificity (SP) of 71%. Furthermore, a positive predictive value (PPV) of 81% was calculated with a corresponding negative predictive value of 81%. The overall diagnostic accuracy was calculated to be 81%. The corresponding ROC curve with an area under the curve (AUC) of 0.86 is presented in Figure 3. Logistic regression performed on the data revealed that including gender, age, smoking (total pack-year), and toombak use increased the predictive probability of the e-nose test measurements to 92.9% (Figure 4).

### 3.3. Blinded Control Group

**Figure 5:**
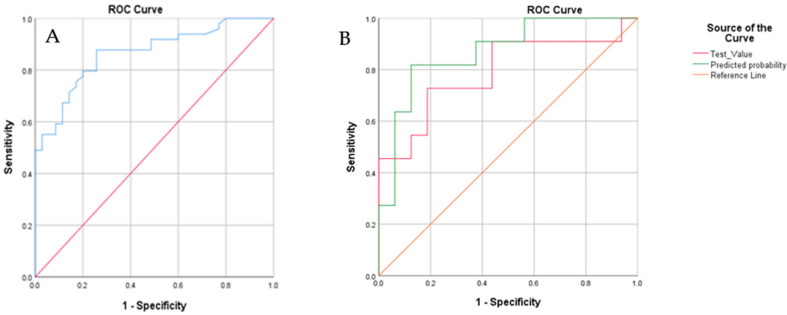
(**A**) The ROC curve for the blind set. The diagonal red line represents the line of no-discrimination, while the blue curve represents different sensitivities and specificities with altered thresholds. The area under the curve is 0.882; (**B**). ROC curves showing the best fit of data for cross-validation when including in the statistical analysis; the clinical parameters of gender, age, smoking (total pack-year), and toombak use (green line; the area under the green curve is 0.92) are compared to the best fit for cross-validation when only the e-nose test values are analyzed (red line).

Some cases were automatically (following the mentioned block size device dependencies rules) assigned to a blinded group by the Aenose software for the validation of the model. Ten patients with OSCC and 17 healthy controls were assigned by the software to this group. The results are as follows: true positive, *n* = 8; true negative, *n* = 13; false positive, *n* = 4; and false-negative, *n* = 2, leading to a PPV of 67% and an NPV of 87%. This shows a sensitivity of 80%, a specificity of 77%, and an accuracy of 79%, which aligns with the values obtained on the validation set. Logistic regression performed on the data for the blind set showed that after the inclusion of the same covariates, i.e., gender, age, smoking (total pack-year), and toombak use, the predictive probability of the e-nose test measurements to 86.9% (Figure 5).

## 4. Discussion

In this study, we reviewed the ability of a portable e-nose to discriminate with an accuracy of 81% between patients diagnosed with OSCC and patients visiting the outpatient clinic for other benign diseases in Sudan, a low-income country in Africa. Of note, the predictability of the e-nose measurements was increased by including gender, age, smoking (total pack-year), and toombak in the statistical model for data analysis, indicating that the ability of e-nose to detect OSCC can be improved even more. Its potential as a diagnostic tool should be further explored on bigger cohorts since more parameters included in the analysis require a higher number of cases available for analysis.

In recent years, the use of VOCs as potential biomarkers for cancer, in general, and HNSCC, in particular, has drawn increasing interest. Gas chromatography-mass spectrometry (GS-MS) has been used in most of the recent research. This technique allows detection of individual VOCs based on their molecular weight. The disadvantages of GS-MS are its high cost, the need for specialized personnel to perform the analysis, and the lack of one specific biomarker for OSCC. Bouza et al. [38] utilized GC-MS methodology and identified several VOCs such as ethanol, 2-propene-nitrile, and undecane dodecane, decanal, benzaldehyde, 3,7-dimethyl undecane, 4,5-dimethyl nonane, 1-octene, and hexadecane as potential biomarkers for the diagnosis of OSCC. Interestingly, they found that butyl acetate was significantly correlated with the histological degree of differentiation. The fact that GS-MS relies on the detection of one single biomarker limits its use as a reliable screening instrument in a clinical setting, particularly in the resource-limited setting of a low-income country such as Sudan. Furthermore, Hakim et al. found an electronic nose containing nanoparticle-based sensors to be superior to GC-MS in distinguishing between HNSCC, lung cancer patients, and healthy controls [39]. Leunis et al. utilized an e-nose with metal-oxide based sensors and confirmed that the resistance patterns of VOCs differed between patients diagnosed with HNSCC and a control group, with a sensitivity of 90% and a corresponding specificity of 80% [32]. Shigeyama et al. [40] identified a signature of 12 VOCs extracted from saliva of OSCC patients as potential OSCC biomarkers. Hartwig et al. [41] confirmed the absence of cancer-associated VOCs in the breath after therapy for HNSCC. However, all these studies on HNSCC including OSCC subsets have been performed on Western populations, in which smoking and alcohol are the major etiological factors and the tongue is the most common site [42]. The present study was performed on a cohort containing a small subset of *toombak*-related OSCC lesions, as indirectly also demonstrated by the preferential buccal and labial localization. Here, we present results indicating that the e-nose might be feasible as a diagnostic tool for populations that have different demographic characteristics and use other types of tobacco than Western populations. The e-nose (Aeonose^™^) used in this study is a handheld and easy-to-use detector. It can be used in areas where high-tech machinery and/or specialized health care workers might not be available, e.g., in rural parts of low-income countries. Therefore, the results of this study provide data to substantiate proposing this device as a feasible solution for OSCC detection in resource-poor areas, such as the rural areas of Sudan, where cancer diagnostic services are unavailable. In principle, to use this device, one would need only a (portable) computer to download the data for calculation, which could be accomplished anywhere in the world where these facilities are available. We believe therefore that in the future, the e-nose might be used as a screening instrument in resource-limited areas where OSCC poses a major burden of disease. We did not perform a special hygienic protocol that would have interrupted the daily routine of the patient. The newer devices can connect to the internet via Wi-Fi and run an unlimited number of validated models with a single measurement. Once further developed and tested, these calibrated models can be easily transferred to an unlimited number of electronic noses. 

## 5. Conclusions

This study shows that e-nose is a feasible technology for detection of OSCC in the Sudanese population, in a cohort with different demographic features than the Western populations previously investigated. It provides further evidence for considering e-nose as a potential tool for early detection of OSCC in resource-limited areas that lack health care infrastructure.

## Figures and Tables

**Table 1:** Demographic and clinical characteristics of the cohort.

Cohort Demographics						
			Non-OSCC Patients		OSCC Patients	
Number of individuals			35		49	
			82.9% males (29)		49% males (24)	
			17.1% females (6)		51% females (25)	
Age *	Males		48.4 years (24–68 years)		55.6 years (21–82 years)	
	Females		33.5 years (27–64 years)		52.2 years (27–80 years)	
Tobacco history and mean pack-years (PY) *			65		35	
Clinical findings for OSCC patients						
Tumor location		Number of cases		Tumor stage		
				Stage		Number (%)
Buccal lower		26.5% (13)		I		2% (1)
Labial lower		24.5% (12)		II		2% (1)
Tongue		14.3% (7)		III		22.5% (11)
Palate		8.2% (4)		IV		63.3% (31)
Other sites		12.2% (6)		Missing staging: 10.2% (5)		
Missing sites data		14.3% (7)				

* Statistically significant differences; *p* < 0.05, *Mann–Whitney U test.*

## Data Availability

The datasets used and/or analyzed during the current study is available as Supplementary Materials as a .csv file.

## Supplementary Materials

The following are available online at https://www.mdpi.com/article/10.3390/healthcare9050534/s1. Supplemenatary Materials 1: Supplementary Material CSV data file e-nose measurements Sudan. Supplemenatary Materials 2: Supplementary Methods—E-nose data anal-ysis flow.
