# Supplementary material for: Feasibility of a Portable Electronic Nose for Detection of Oral Squamous Cell Carcinoma in Sudan
Source: Healthcare (Basel). 2021 May 3;9(5):534. doi: 10.3390/healthcare9050534 (PMC8147635; doi:10.3390/healthcare9050534)
Supplement: Supplementary file 1 [file healthcare-09-00534-s001.zip › Supplemenatary Materials 2-Supplementary Methods - E-nose data analysis flow.pdf]

The data process flow pursued was as follows:

1. Starting from raw data (.csv file supplementary material)

a. Normalize all datapoints per participant between 0 and 1.

b. Apply peak shaving to remove spikes

c. Apply Fourier transform and compensate for clean air signal

d. Apply Fourier back transform

e. Apply e-power to all data points

2. Select NOx-sensor only

3. Apply feature extraction to end up with a 19-element vector per participant

4. Normalize the vectors between -1 and +1

5. Apply a resilient backpropagating artificial neural network for training using the following parameters:

a. Max Epoch : 5000

b. Max Retries : 25

c. Max Same Error : 30

d. Max Error Inc : 15

e. Minimal Error : 0.0005

f. Learn Rate : 0.0010

g. Alpha : 0.0500

h. Topology : 17x7

6. ROC-curve is obtained when applying ‘Leave-10%-out’ cross validation on the datapoints.
